# Development of a Primary Care Cardio-Kidney Risk Navigator for Clinical Decision Support

**DOI:** 10.3390/jcm15145379

**Published:** 2026-07-09

**Authors:** Tyler J. Gluckman, Kade Birkeland, Ankeet Bhatt, Matthew Jay Budoff, Samuel Colby Danna, Nihar R. Desai, Evan Norfolk, Ahlam Elbedewe, Grace Kiernan, Radha Pachpor, Jamie Hirsch

**Affiliations:** 1 Center for Cardiovascular Analytics, Research, and Data Science (CARDS), Providence Heart Institute, Providence Health System, Portland, OR 97225, USA; 2 Research Data Services, Cedars-Sinai Medical Center, Los Angeles, CA 90048, USA; 3 Division of Research, Kaiser Permanente San Francisco Medical Center, San Francisco, CA 94588, USA; 4 Division of Cardiology, Harbor-UCLA Medical Center, The Lundquist Institute for Biomedical Innovation at Harbor-UCLA Medical Center, Torrance, CA 90502, USA; 5 Department of Primary Care, Veterans Southeast Louisiana Healthcare System, New Orleans, LA 70119, USA; 6 Section of Cardiovascular Medicine, Yale School of Medicine, Yale University, New Haven, CT 06520-8017, USA; 7 Department of Nephrology, Geisinger Health, Danville, PA 17822, USA; 8 Care Delivery, Petauri Kinect, New York, NY 10001, USA; grace.kiernan@petauri.com (G.K.); radha.pachpor@petauri.com (R.P.); 9 Department of Diabetes and Cardiometabolic Health, Premium Health, Brooklyn, NY 11201, USA

**Keywords:** chronic kidney disease, cardiovascular disease, clinical pathways, primary care, screening, management, quality improvement, risk stratification

## Abstract

**Background/Objectives**: Chronic kidney disease (CKD) and cardiovascular disease (CVD) confer a substantial, interrelated health burden. CKD markedly increases cardiovascular risk and premature mortality, while CVD accelerates kidney disease progression. Despite the availability of simple diagnostic tests and validated CVD risk tools, CKD screening remains inconsistent, kidney measures are under-integrated into CVD risk stratification, and guideline-recommended screening is variably adopted due to system-, clinician-, and patient-level barriers. This initiative aimed to develop a consensus-based, practical algorithm to integrate CKD screening into CVD risk management. **Methods**: A structured Delphi process was used to develop an evidence-informed, consensus-based screening algorithm. In Phase 1, a multidisciplinary expert panel completed iterative surveys to identify and prioritize key screening components and achieve preliminary consensus. Participants received a landscape assessment summarizing current practices, evidence, and implementation gaps. A roundtable discussion reviewed clinical guidelines, published evidence, and survey findings, informing the development of an initial algorithm draft. In Phase 2, a follow-up roundtable refined the algorithm, assessed feasibility within real-world clinical workflows, and confirmed consensus on priority elements. Agreement was finalized through structured group discussion and survey-based validation. **Results**: Panel discussions identified optimal CKD screening triggers, key gaps in current practice, and opportunities to promote earlier identification of at-risk patients. Refinement during the second roundtable resulted in consensus on algorithm structure, content, and applicability across settings. The final algorithm reflects a streamlined, implementation-focused approach to support consistent and earlier identification of at-risk patients. **Conclusions**: The algorithm, labeled the Primary Care Cardio-Kidney Risk Navigator, provides a practical, flexible framework that integrates and operationalizes existing guideline recommendations into a unified, workflow-oriented approach for primary care that supports consistent real-world implementation. It supports earlier identification, referral, and prevention strategies for at-risk populations and can be implemented within electronic health records or as a standalone clinical decision support tool.

## 1. Introduction

Chronic kidney disease (CKD) and cardiovascular disease (CVD) are among the most significant public health challenges worldwide. In the United States, CKD affects more than 35 million adults, and most remain undiagnosed until later disease stages [1]. Costs of CKD increase as the disease progresses, rising by as much as 46% per person per year between stage 3 and stages 4–5 [2]. Early identification is critical because kidney dysfunction not only leads to increased costs but also drives excess cardiovascular and all-cause mortality [3].

The burden of CVD is even higher than that of CKD, affecting approximately 127.9 million adults in the United States [4]. More than USD 400 billion is spent on direct (e.g., medical services) and indirect (e.g., lost productivity) costs of CVD annually, with direct costs accounting for 11% of total US health expenditures [4]. Beyond its population-level burden, CVD and CKD are closely interconnected, with extensive evidence demonstrating a bidirectional relationship in which CKD increases cardiovascular risk, and established CVD, in turn, accelerates kidney disease progression through shared pathophysiologic mechanisms, including hemodynamic stress, inflammation, and neurohormonal dysregulation [5].

The growing clinical and economic burden of both CKD and CVD underscores the urgent need for early screening and diagnosis. CKD screening can identify early loss of renal function and enable interventions that delay its progression and limit adverse outcomes, but systematic implementation is uneven, and many high-risk patients do not receive complete testing, particularly in primary care settings [6]. Current CKD guidelines issued in 2024 by Kidney Disease: Improving Global Outcomes (KDIGO) recommend blood (i.e., estimated glomerular filtration rate [eGFR]) and urine (i.e., urine albumin-to-creatinine ratio [uACR]) tests for primary screening of CKD. To assess CVD risk, contemporary approaches to cardiovascular risk assessment, including pooled cohort equation-based atherosclerotic cardiovascular disease (ASCVD) risk estimation and the Predicting Risk of CVD EVENTs (PREVENT™) equations, recommend broad implementation of CVD screening through assessment of blood pressure, cholesterol levels, and validated risk-scoring tools [7,8].

However, recent analyses indicate that existing methods may miss a substantial proportion of individuals at true risk, highlighting limitations in current screening paradigms for CVD [9]. Addressing these gaps requires coordinated, interdisciplinary approaches that align screening, risk assessment, and management across primary care and specialty settings.

Consistent implementation of CKD and CVD screening guidelines is impeded by multiple barriers, including underutilization of uACR and eGFR testing, gaps in guideline awareness, and systemic delays that undermine early detection and underestimate CV risk [9,10]. In particular, low rates of uACR testing among high-risk groups (e.g., individuals with diabetes or hypertension) are well documented, with negative impacts on the cost, timeliness, and quality of care [9,10].

For many patients, there is an overlap of cardiovascular, kidney, and metabolic risk factors, known as cardiovascular-kidney-metabolic (CKM) syndrome. The CKM framework includes interconnected risk factors that collectively increase CVD risk, such as hypertension, diabetes, dyslipidemia, obesity, and CKD [11]. The framework describes progressive CKM stages ranging from the absence of risk factors to established CVD [12]. Moreover, CKD is a potent and independent risk factor for CVD, and it elevates the risk of coronary artery disease, heart failure, arrhythmias, and sudden death [13]. As a result, the risk of cardiovascular death exceeds the risk of progression to end-stage renal disease among patients with CKD, underscoring the critical need for integrated risk prediction tools that account for kidney function measures such as eGFR and albuminuria, as well as CVD risk [14]. This effort aimed to develop a consensus algorithm that incorporates available guidelines and literature, as well as recommendations from a panel of experts in primary care, nephrology, cardiology, and a pharmacist with informatics expertise, to support primary care clinicians in using a multimodal approach to identify CKD within the broader context of CVD risk. CKM disease, encompassing CKD, CVD, and other conditions, represents a major and growing challenge in primary care, as its interconnected nature necessitates integrated, patient-centered management and coordinated, interdisciplinary care involving primary care, cardiology, nephrology, and endocrinology to address fragmentation in clinical practice, which contributes to gaps in early detection and risk management [15].

## 2. Methods

A structured Delphi technique was employed to develop a consensus-based algorithm for integrated CKD screening within CVD risk management. Development of the consensus algorithm was guided by an advisory panel that included participants from the Heart Health Leaders Network and the CKD Leaders Network (Table 1). In advance of the initial roundtable discussion, experts were provided with a landscape assessment as a pre-read to contextualize current screening practices, implementation considerations, and gaps in care.

The first roundtable meeting was held on 12 August 2025. Advisors participated in an iterative survey to gain their perspectives on screening practices and key components, and achieve preliminary consensus on screening priorities, including patient identification and screening triggers; screening modalities and data inputs; design, implementation, and workflow; and prioritization and evidence gaps. Participants then reviewed and discussed current CKD and CVD clinical guidelines (Table 2) and evidence, ensuring alignment with best practices. Insights from this discussion informed the creation of an initial draft of the algorithm.

Following Roundtable 1, the Primary Care Cardio-Kidney Risk Navigator, a clinical decision support (CDS) algorithm for integrated CKD screening in CVD risk management, was drafted. The algorithm was then reviewed by the roundtable panel and modified based on their feedback.

The algorithm encompasses the Roundtable 1 survey results, meeting output, and published CKD and CVD guidelines from KDIGO, AHA/American College of Cardiology (ACC), ADA, and CDC CKD Screening Guidance. It categorizes patients by CKM stages (0–4), consistent with the AHA CKM staging framework [12]. It is then organized into 4 phases of management: (1) Screening and Data Collection, (2) Risk Stratification, (3) Management and Referrals, and (4) Follow-Up and Monitoring. Care settings include primary care, cardiology, nephrology, endocrinology, and multidisciplinary support. Each step within the model can be expanded to better understand additional details.

In the second phase, a follow-up roundtable meeting was held on 13 January 2026 to refine the algorithm, validate its applicability in clinical workflows, and confirm agreement on priority elements. Consensus was finalized through structured individual and group discussions, resulting in an evidence-informed algorithm designed to support integrated CKD screening in patients at risk for CVD. This structured, multi-phase process ensured that the resulting algorithm reflects both current evidence and real-world clinical expertise, with a focus on feasibility and applicability across diverse practice settings.

The Delphi process followed a structured, multi-step approach to achieve consensus across expert participants. In Round 1, panelists completed a survey designed to assess current screening practices, identify key gaps, and prioritize components for inclusion in the proposed algorithm. Survey items included Likert-scale ratings and structured ranking questions focused on patient identification, screening triggers, data inputs, workflow integration, and implementation considerations. The expert panel included clinicians representing primary care, cardiology, nephrology, and pharmacy perspectives, with majority participation maintained across both roundtable discussions to ensure continuity of input.

Survey findings were aggregated and used to guide discussion during the initial roundtable meeting, where areas of alignment and divergence were reviewed. Insights from this discussion informed the development of the preliminary algorithm. In Round 2, the revised framework was presented to participants for further refinement, with emphasis on clinical accuracy, usability, and feasibility within real-world workflows.

Consensus was established through the convergence of expert opinions during structured discussion and confirmation of agreement across core elements during the second roundtable. This iterative process ensured that the final algorithm reflects both evidence-informed recommendations and practical considerations for implementation in primary care.

## 3. Results

### 3.1. Roundtable 1

#### 3.1.1. Roundtable 1 Discussion

The first roundtable identified high-priority needs to guide the development of an algorithm to improve integrated CKD and CVD screening:

- Early identification of high-risk patients;
- Standardized patient-clinician education;
- Clear referral triggers and shared accountability;
- Identification of additional gaps or emerging priorities.

The panel identified the top five CVD-relevant triggers for CKD screening in primary care as diabetes, hypertension, an abnormal eGFR, an elevated uACR, and a history of CVD (e.g., myocardial infarction, stroke). Although the panel agreed that uACR testing should be straightforward for clinicians to order, electronic medical record (EMR)-driven workflow complexity and lack of awareness were cited as primary barriers contributing to suboptimal uACR testing, alongside limited automation and over-reliance on individual clinicians. The top five primary triggers for CVD screening were diabetes, hypertension, elevated low-density lipoprotein (LDL) or total cholesterol, smoking history, and obesity (BMI ≥ 30), with or without waist circumference.

**Figure 1:**
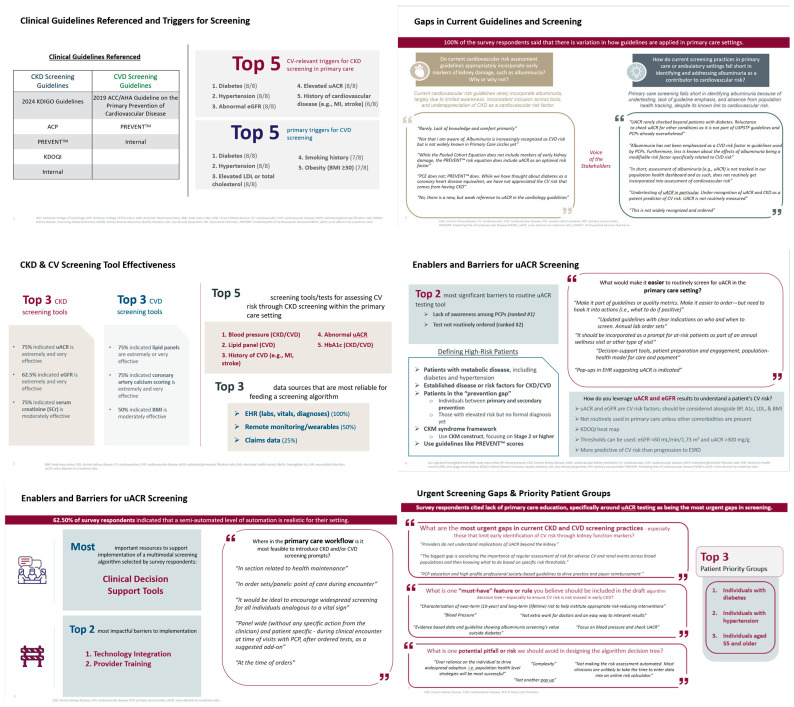
Roundtable 1 survey results.

Survey results (Figure 1) identified a lack of primary care education—particularly regarding uACR testing—as the most urgent gap in CKD/CVD screening. All respondents also reported substantial variation in how clinical guidelines are applied in primary care. Current cardiovascular risk guidelines inadequately incorporate albuminuria, likely due to limited clinician awareness, inconsistent inclusion across risk assessment tools, and underappreciation of CKD as a cardiovascular risk factor. As a result, primary care screening often fails to identify albuminuria because of undertesting, limited emphasis in guidelines, and exclusion from population health tracking, despite its well-established association with cardiovascular risk.

#### 3.1.2. Roundtable 1 Recommendations

To address these challenges, the panel agreed upon the following recommendations:

1. Make uACR Testing Routine in Primary Care. Elevate uACR to be a key test alongside eGFR, low-density lipoprotein cholesterol (LDL-C), and blood pressure for clinicians to initiate targeted therapy earlier, especially for cardio-kidney risk amplification. Embed a one-click lab bundle that includes uACR and eGFR in annual diabetes and hypertension order sets and auto-surface “overdue” status in a non-interruptive care-gap panel, while pairing with a brief, patient-facing explanation.
2. Integrate CKD Markers and Apply the PREVENT^TM^ Equations for Risk Assessment. Use uACR and eGFR to reclassify patient risk, while applying the PREVENT^TM^ risk equations and the KDIGO prognosis of CKD by eGFR and albuminuria categories, color-coding [3] to clarify treatment intensity. Enable the PREVENT^TM^ risk equations in the EMR, display the KDIGO category next to the patient’s risk score, and use uACR results to adjust therapy intensification. If the PREVENT^TM^ risk equations are unavailable within the EMR, they can be accessed online: https://professional.heart.org/en/guidelines-and-statements/prevent-calculator, accessed on 10 May 2026). Alternatively, it is reasonable to use the pooled cohort equation (PCE) with a “CKD risk enhancer,” flagging patients with CKD markers (e.g., uACR, eGFR) to indicate higher-risk patients (beyond those identified by the PCE alone), aligning with KDIGO and ACC/AHA recommendations.
3. Incorporate GDMT Into Protocols to Enable Team-based Care. Standardize stakeholder responsibilities and automate orders/titrations to close gaps faster while reducing primary care provider (PCP) burden. Consider focusing on modifiable risk factors such as uACR, LDL-C, blood pressure, and HbA1c. Leverage existing protocols that start with an angiotensin-converting enzyme (ACE) inhibitor or angiotensin receptor blocker (ARB), by adding an SGLT2 inhibitor and statin, followed by a mineralocorticoid receptor antagonist (MRA) and/or glucagon-like peptide-1 receptor agonist (GLP-1 RA), supported by pharmacist-led titration. For example, have the pharmacy run weekly titration lists, with PCP review only by exception. Recheck the uACR and eGFR within 3 to 6 months after any therapy change.
4. Increase Automation of Education. Convert guidelines into workflow-embedded defaults, enabling education to be delivered through the EMR with minimal clicks. Replace interruptive alerts with a care-gap panel showing labs/medications due, enable orders to be signed with a single click, and provide in-context tooltips (e.g., “Why uACR?”) instead of separate trainings.
5. Streamline Patient Education. Reduce the variability and time required for education by offering it in multiple formats (electronic health record [EHR] portal and printed), triggered automatically with orders and results. Create a simple patient journey: order a uACR, automatically send an explanation (“What is uACR?”), share results with a clear risk tier, and, if elevated, provide medication information with a follow-up plan.
6. Establish Clear Referral Triggers for Shared Accountability Between Specialty Teams. Codify referral criteria and co-management lanes so elevated uACR/eGFR risk triggers the right specialty consult at the right time. Establish EMR order sets with referral triggers to nephrology for severe albuminuria, rapid kidney function decline, or hard-to-control blood pressure, and to cardiology for high cardiovascular (CV) risk, early CKM disease, or persistent high LDL-C despite treatment.
7. Pilot the Algorithm in Diabetes. Gain early buy-in by proving feasibility in diabetes—a disease state where guidelines and workflows already support testing. Thereafter, scale to hypertension, ASCVD, and CKD stage ≥3 once barriers are known. Pre-agree on metrics (e.g., uACR and eGFR patient education completion) and consider implementing an 8- to 12-week pilot in 1 or 2 clinics. The findings can then be used to justify expansion.
8. Deployment Efforts Should Leverage Implementation Science Principles. Define the details of what an implementation pilot could entail, establish metrics and barriers, and leverage a CDS toolkit that others can adopt. Carry forward best practices and iterations to the workflow by keeping a log of barriers (e.g., information technology, workflow, patient communication) and updating the pathway before scaling.
9. Ensure Actionable Results When Building CDS Tools. Avoid complexity and design EMR solutions that minimize clicks (e.g., bundled orders, “Select All” default, care-gap panels vs. alerts) to maximize uptake and minimize fatigue. Create standardized order sets for diabetes care that include key labs (e.g., HbA1c, kidney function, cholesterol), preselected for easy ordering with a quick sign-off and built-in checks to avoid duplicate tests.
10. Prepare for Artificial Intelligence (AI). While AI tools are not yet ready for widespread implementation, today’s value lies in simple, scalable steps. Streamline relevant data; ensure uACR and eGFR are measured regularly, and that medications and vitals are recorded in structured fields to support future AI tools. Standardize coding and flowsheets now, track data completeness as a key performance indicator, and pilot low-risk AI assistants later (e.g., identify patients with missing uACR who resemble high-risk cohorts).

### 3.2. Roundtable 2

#### Roundtable 2 Discussion

**Figure 2:**
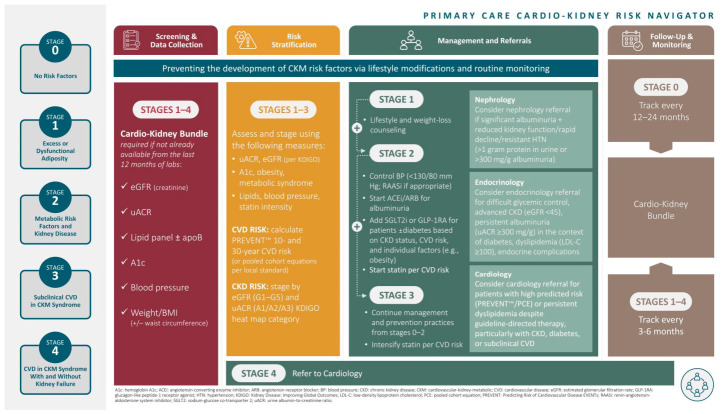
Primary care cardio-kidney risk navigator [8,11].

Building on consensus from Roundtable 1, the second roundtable advanced development of a primary care-oriented Cardio-Kidney Risk Navigator (Figure 2) by refining the decision-tree algorithm, clarifying the target patient population and workflow sequence, and prioritizing operational simplicity to support realistic adoption in primary care. Participants agreed that the Navigator should function as a guideline-based care pathway and implementation blueprint, providing recommended sequencing for assessment, risk stratification, and escalation while reflecting real-world feasibility across diverse health system archetypes. The tool was envisioned to be EMR-build-ready, but not dependent on EMR development to be useful, supporting flexibility across practice environments.

Much of the discussion centered on the identification of at-risk individuals. Several experts recommended a clear patient type triage to mirror how clinicians think about CKM risk, segmenting patients into different groups: those with risk factors only, those with established CKD or metabolic disease, and those with established CVD or for whom further estimation of cardiovascular risk is not needed. The group confirmed that uACR and eGFR testing should remain foundational, while keeping emphasis on streamlining bundled orders and automating default behavior, consistent with Roundtable 1 insights.

To strengthen risk stratification and referral logic, feedback focused on improving clarity and ensuring clinical accuracy, with consistent terminology used for referral thresholds. The experts recommend that referral to endocrinology occur only in cases where their involvement provides a clear, meaningful contribution beyond what primary care or nephrology can address (e.g., advanced CKD with insulin management risk). They cautioned that redundant PREVENT^TM^ calculations should be avoided for patients with CVD and suggested specific numeric cutoffs for risk-enhancing factors aligned with ACC/AHA guidance.

The group aligned on specialty roles across CKM care to remove ambiguity and prevent misdirected referrals. Cardiology involvement should focus on risk-enhancing factors, lipid management targets, and CVD-related intensification. Nephrology referral triggers should focus on severe albuminuria, rapid eGFR decline, resistant hypertension, uncertainty in CKD etiology, or complexity beyond the primary care scope. Endocrinology involvement is narrower and primarily tied to glycemic management challenges, especially insulin titration in patients with a low eGFR. Furthermore, the group emphasized that specialty involvement should occur through coordinated co-management with primary care and other disciplines, rather than in isolated siloes, to ensure continuity and alignment of CKM care.

Additional refinements focused on aligning the framework with CKM-specific staging to ensure consistent risk stratification across care settings, while clearly defining referral pathways to support timely, appropriate specialty involvement. These refinements are intended to enhance usability in routine clinical practice by aligning the algorithm with how primary care clinicians structure patient evaluation and management decisions, to further enhance clarity and practicality for primary care implementation, and to facilitate seamless care transitions across the CKM continuum.

## 4. Discussion

The Primary Care Cardio-Kidney Risk Navigator provides a structured CDS framework that integrates multiple guideline recommendations into a workflow-oriented approach for primary care. This approach enables earlier identification, appropriate referral, and more effective treatment and prevention strategies for at-risk populations. The tool is intended to help primary care clinicians identify cardiometabolic risk earlier, collect the right foundational data, and apply guideline-based next steps for patients across all CKM stages, whether they present with no risk factors or already have established cardiovascular or kidney disease. By streamlining patient identification and referral workflows, the Navigator supports improved quality of care while promoting more efficient use of health system resources.

This work does not introduce new screening modalities but rather focuses on translating and operationalizing existing evidence into a cohesive, implementation-ready clinical framework. Although CKD screening tests and risk factors have been well established, their consistent application in primary care remains suboptimal. This work addresses the critical gap between guideline recommendations and real-world practice by integrating CKD and CVD screening into a unified model that reflects how clinicians make decisions in routine care. The Cardio-Kidney Risk Navigator advances prior approaches by embedding screening triggers, risk stratification, referral pathways, and follow-up processes within a structured, end-to-end clinical workflow designed for scalability across diverse healthcare settings.

While individual components of CKD and CVD screening are well established, their application in routine care is often fragmented and inconsistently operationalized. This work contributes by consolidating existing recommendations into a structured, workflow-oriented model that reflects how screening, risk stratification, and referral decisions occur in practice. The Navigator organizes guideline-aligned elements into a sequenced pathway with defined triggers, integrated decision points, and coordinated referral considerations, offering a practical approach to reducing variability in implementation across care settings. In this way, the framework supports more consistent application of existing evidence within real-world primary care workflows.

In practice, the Navigator can be applied either as a standalone clinical framework or integrated into EMRs. In settings without advanced digital infrastructure, the framework can guide clinical decision-making independently. Alternatively, when integrated into EMRs, it can be operationalized through bundled laboratory orders, care-gap panels, and structured risk-stratification tools. Key workflow steps include the identification of at-risk patients, completion of recommended screening tests, risk stratification using validated tools, initiation of GDMTs-, and referral and follow-up monitoring as appropriate. This structured sequencing reflects common clinical decision pathways and is intended to align with how care is delivered in routine practice.

A critical consideration for implementation is variability in access to recommended screening modalities, particularly uACR testing. Although uACR is a foundational component of CKD detection and risk stratification, access may be limited in certain practice environments, including rural or resource-constrained settings where laboratory infrastructure or workflow integration may be inconsistent. In these contexts, implementation strategies may require adaptation, such as prioritizing opportunistic screening, leveraging simplified testing pathways, or adopting phased implementation approaches aligned with local resource capabilities. Ensuring that the Navigator can function in both EMR-integrated and standalone formats may support broader uptake across diverse care settings.

Patient adherence to follow-up testing, monitoring, and referrals is another important determinant of real-world effectiveness. Even when screening is completed, gaps in follow-up, such as delayed repeat testing, incomplete referrals, or limited patient understanding, may reduce the impact of early identification efforts. Prior work from the roundtables emphasized the importance of streamlined patient education, automated communication, and clearly defined care pathways to support engagement across the care continuum. Embedding education at the point of care, simplifying the patient journey, and aligning referral pathways with clear accountability across specialties may help mitigate these challenges. Additionally, integrating follow-up tracking within workflows, such as through care gap panels or standardized monitoring intervals, may improve continuity and reduce missed opportunities for intervention. To support practical implementation, the Navigator is designed to align with common primary care workflows. Screening may be initiated during annual preventive visits, chronic disease follow-up encounters (e.g., diabetes or hypertension management), or opportunistically when relevant laboratory or clinical data become available. The primary target population includes adults with established CVD, diabetes, hypertension, or other cardiometabolic risk factors, as well as individuals identified through abnormal laboratory findings such as reduced eGFR or elevated uACR.

A key limitation of this work is that the Primary Care Cardio-Kidney Risk Navigator has not yet undergone prospective validation in real-world clinical settings, as this effort focused on consensus development and framework design. As such, this work is intended to serve as a foundation for future implementation and evaluation rather than a validated clinical intervention. Future work should focus on pilot implementation studies across diverse healthcare settings to assess feasibility, adoption, and effectiveness, followed by prospective validation to determine its impact on CKD and CVD screening rates, risk stratification accuracy, and downstream clinical outcomes.

Looking toward the future, emerging methods hold promise for earlier detection, individualized risk stratification, and monitoring of CKD and CVD progression. Investigative biomarkers may offer enhanced sensitivity and specificity for early detection of kidney damage, disease progression monitoring, and risk stratification, and machine learning models and AI-enhanced tools may augment traditional risk assessment approaches. As these methods become more widely available, they may be incorporated into the Navigator for continued decision support to facilitate guideline-based practices and optimize care of patients with CKD and/or CVD.

## 5. Conclusions

In summary, the Primary Care Cardio-Kidney Risk Navigator represents a consensus-driven, evidence-informed CDS framework designed to address persistent gaps in integrated CKD and CVD screening within primary care. By aligning contemporary guideline recommendations with real-world workflow considerations, the Navigator provides a practical approach to earlier identification of at-risk individuals, improved risk stratification, and more consistent application of guideline-directed management across the CKM continuum. Its emphasis on operational simplicity, standardized triggers, and clarified referral pathways supports scalability across diverse health system environments. This workflow-oriented design enables seamless integration into routine primary care processes, supporting real-time decision-making and consistent application of screening and management strategies at the point of care. Importantly, the Navigator is designed to function both as an EMR-embedded tool and as a standalone implementation blueprint, enabling broad adoption regardless of local digital maturity. As healthcare systems increasingly seek to align cardiovascular, kidney, and metabolic risk management, this framework offers a foundational model to support quality improvement efforts, close screening gaps, and ultimately improve outcomes for patients with or at risk for CKD and CVD. Future research should focus on pilot implementation and longitudinal evaluation to assess feasibility, uptake, and clinical effectiveness, with the goal of refining the model and supporting wider dissemination. As healthcare systems increasingly move toward integrated cardiometabolic care, this framework provides a foundational approach to improving early detection, coordination, and outcomes for patients with or at risk for CKD and CVD.

## Figures and Tables

**Table 1:** Roundtable participant list.

Name	Title	Institution
Ankeet Bhatt, MD, MBA, ScM	Cardiologist and Clinical Investigator	Kaiser Permanente Northern California San Francisco, CA, USA
Evan Norfolk, MD	Division Director and Chief of Nephrology	Geisinger Health Danville, PA, USA
Jamie Hirsch, MD *	Nephrologist and Associate Vice President Data Science and Predictive Analytics	Northwell Health New Hyde Park, NY, USA
Kade Birkeland, PharmD ^†^	Manager, Research Data Services	Cedars-Sinai Medical Center Los Angeles, CA, USA
Matthew Jay Budoff, MD, FACC, FAHA	Endowed Chair of Preventive Cardiology and Program Director	The Lundquist Institute for Biomedical Innovation at Harbor-UCLA School of Medicine Torrance, CA, USA
Nihar R. Desai, MD, MPH	Vice Chief and Associate Professor (Cardiovascular Medicine)	Yale School of Medicine New Haven, CT, USA
Samuel Colby Danna, MD	Deputy Chief of Ambulatory and Primary Care	Veterans Healthcare System New Orleans, LA, USA
Tyler J. Gluckman, MD, MHA	Medical Director, Center for Cardiovascular Analytics, Research, and Data Science (CARDS)	Providence Heart Institute, Providence Health System Portland, OR, USA

* Moved to a new role as Nephrologist and Medical Director of Diabetes and Cardiometabolic Health at Premium Health, Brooklyn, New York, after roundtable discussions. ^†^ Participated in Roundtable 1 only.

**Table 2:** Guideline sources for CKD/CVD screening parameters.

Screening Parameter	Details	Source(s)
uACR and eGFR as Risk Stratification Tools	UACR and eGFR are core markers for CKD classification, risk stratification, and cardiovascular risk assessment. The KDIGO heat map integrates eGFR and albuminuria categories to define CKD prognosis and guide management intensity. Reduced eGFR (<60 mL/min/1.73 m^2^) and elevated uACR (≥30 mg/g) are diagnostic markers of CKD and are associated with increased cardiovascular risk.	KDIGO2024 Guidelines [3]
PREVENT™ Risk Equation	Incorporates uACR, eGFR, hemoglobin A1c (HbA1c), and body mass index (BMI) for 10- and 30-year CVD risk modeling. Developed as a next-generation risk tool that expands beyond traditional pooled cohort equations to incorporate cardiovascular, kidney, and metabolic risk.	American Heart Association (AHA) PREVENT^TM^ Equations (2024) [8]
Guideline-Directed Medical Therapy (GDMT)	Recommend sodium-glucose co-transporter 2 (SGLT2) inhibitors, renin-angiotensin system (RAS) inhibitors, and statins for CKD patients with cardiovascular risk. Emphasize protocolized therapy and team-based care.	KDIGO 2024 Guidelines [3]; American Diabetes Association (ADA) Standards of Care 2025 [16]
CKD Screening Recommendations	Recommend targeted CKD screening in individuals at high risk, including those with diabetes, hypertension, or CVD.	Centers for Disease Control and Prevention (CDC) CKD Screening Guidance [1] US Preventive Services Task Force Status: No current recommendation for routine CKD screening; topic under review

## Data Availability

Supporting materials are available through the CKD Leaders Network and Heart Health Leaders Network on the Networks of Excellence website (https://www.networksofexcellence.org/, accessed on 1 June 2026), subject to governance and privacy considerations.

## Abbreviations

The following abbreviations are used in this manuscript:

A1c	Hemoglobin A1c
ACE	Angiotensin-Converting Enzyme
ACC	American College of Cardiology
AHA	American Heart Association
AI	Artificial Intelligence
ARB	Angiotensin Receptor Blocker
ASCVD	Atherosclerotic Cardiovascular Disease
BMI	Body Mass Index
CDC	Centers for Disease Control and Prevention
CDS	Clinical Decision Support
CKD	Chronic Kidney Disease
CKM	Cardiovascular-Kidney-Metabolic
CVD	Cardiovascular Disease
eGFR	Estimated Glomerular Filtration Rate
EMR	Electronic Medical Record
GDMT	Guideline-Directed Medical Therapy
GLP-1 RA	Glucagon-Like Peptide-1 Receptor Agonist
KDIGO	Kidney Disease: Improving Global Outcomes
LDL	Low-Density Lipoprotein
LDL-C	Low-Density Lipoprotein Cholesterol
MI	Myocardial Infarction
MRA	Mineralocorticoid Receptor Antagonist
PCP	Primary Care Provider
PCE	Pooled Cohort Equation
PREVENT^TM^	Predicting Risk of CVD EVENTs (risk equations)
SGLT2	Sodium-Glucose Co-transporter -2
uACR	Urine Albumin-to-Creatinine Ratio
